# Two Wire System and Modified Olive Tip to Facilitate Implantation of Fenestrated TEVAR in Patient with Proximal Descending Aortic Pathology: First Two Cases

**DOI:** 10.1007/s00270-019-02183-z

**Published:** 2019-02-14

**Authors:** Suko Adiarto, Sung Gwon Kang, Ismoyo Sunu, Taofan Siddiq, Hananto Andriantoro, Iwan Dakota, Raman Uberoi

**Affiliations:** 1 Department of Cardiology and Vascular Medicine, Universitas Indonesia/Harapan Kita National Cardiovascular Center, Jl. Letjend S Parman kav. 87 Slipi, 11420 Jakarta, Indonesia; 2 Department of Radiology, Chosun University, 365 Pilmundaero, Dong-gu, Gwangju-Si 61453 South Korea; 3 Department of Radiology, John Redcliffe Hospital, Oxford, OX3 9DU UK

**Keywords:** Proximal descending aortic pathology, Fenestrated TEVAR, Two wires system

## Abstract

**Introduction:**

Although Fenestrated TEVAR (F-TEVAR) has been considered to be a more physiologic approach to treat proximal descending aortic pathology, its application is still limited due to availability, cost and technical difficulties. We introduce a new design of fenestrated stent graft with a new delivery system and successfully performed first in human implantation in two patients, one with an aortic aneurysm and one with an acute aortic dissection.

**Materials and Methods:**

The design of these two wires fenestrated stent graft include creation of an additional lumen at the side of the olive tip during manufacture, from which an additional wire can be introduced for a side branch passing into the fenestration, running inside the stent graft and exit the delivery sheath through additional hub. The two wires will facilitate delivery and deployment of the stent graft. One patient with descending aortic aneurysm and another with Stanford B aortic dissection is included in this first in human study.

**Results:**

The aneurysm and dissection were completely excluded immediately after the TEVAR. Six month follow up CT showed good position of the stent graft and patent LSA in both patients. In the patient with aortic dissection, expansion of the true lumen and partial thrombosis of the false lumen was seen.

**Conclusions:**

This is a report of a two wire system in 2 patients with distal aortic arch pathology demonstrating a good technical and clinical success using pre-cannulated fenestrations through a modified nose cone olive.

**Level of Evidence:**

Level 4, report of two cases.

## Introduction

Thoracic endovascular aortic repair (TEVAR) has emerged as an effective alternative to open surgical repair for the treatment of descending aortic aneurysms and dissections. Over the last 20 years, the clinical outcome of TEVAR has been consistently superior to that of open surgical repair [1–4]. However, its application has been limited to lesions with landing zone of > 1.5–2 cm [5]. For those with inadequate landing zones, TEVAR can still be performed by sacrificing the left subclavian artery (LSA), performing bypass surgery, using Chimney techniques or by implanting branched/fenestrated stent grafts [6, 7].

However all these techniques have their drawbacks and fenestrated or branched stent grafts would seem to be a more physiological approach. The majority of these devices have to be customized however and their availability as well as costs remain a major concern [8–10]. Furthermore, positioning and deployment are still considered difficult [11, 12]. In this study, we report on a new design of a fenestrated stent graft with a novel modification of the olive tip and delivery system with its first application in two patients.

## Materials and Methods

### Modified Stent Graft (Fig. 1)

**Fig. 1:**
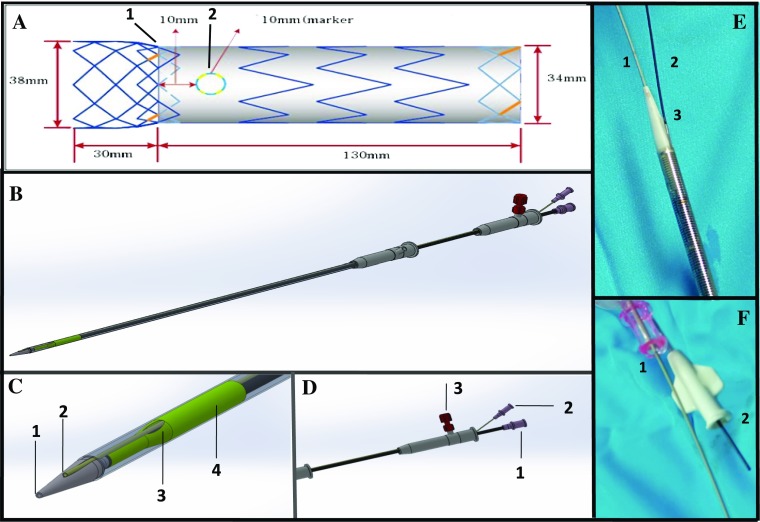
Schematic design of the fenestrated stent graft with two wire system. **A** Schematic figure of the Fenestrated stent graft. **B** The whole length of the stent graft and delivery system. **C** Proximal part of the system: (1) Central hole of the olive tip, from which the super stiff wire is inserted, (2) Side hole of the olive tip. The side branch wire that is positioned in the left LSA is inserted to the delivery system from this hole, (3) Fenestration hole of the stent graft and (4) Stent graft. **D** Distal part of the system: (1) Central hub, from which the central wire (stiff wire) will come out of the delivery system, (2) Side hub, from which the side wire will come out of the delivery system and (3) Delayed deployment system. **E** Photograph of Proximal part of the system: (1) Lunderquist guide wire loaded to central hole of the olive tip, (2) Hydrophilic guide wire loaded to side hole of the olive tip and (3) The small tube connecting the side hole of the olive tip and the side hub at the end of delivery sheath. **F** Photograph of Proximal part of the system: (1) Lunderquist guide wire exit the delivery sheath through the central hub and (2) Hydrophilic guide wire exit the delivery sheath through the side hub

**Fig. 2:**
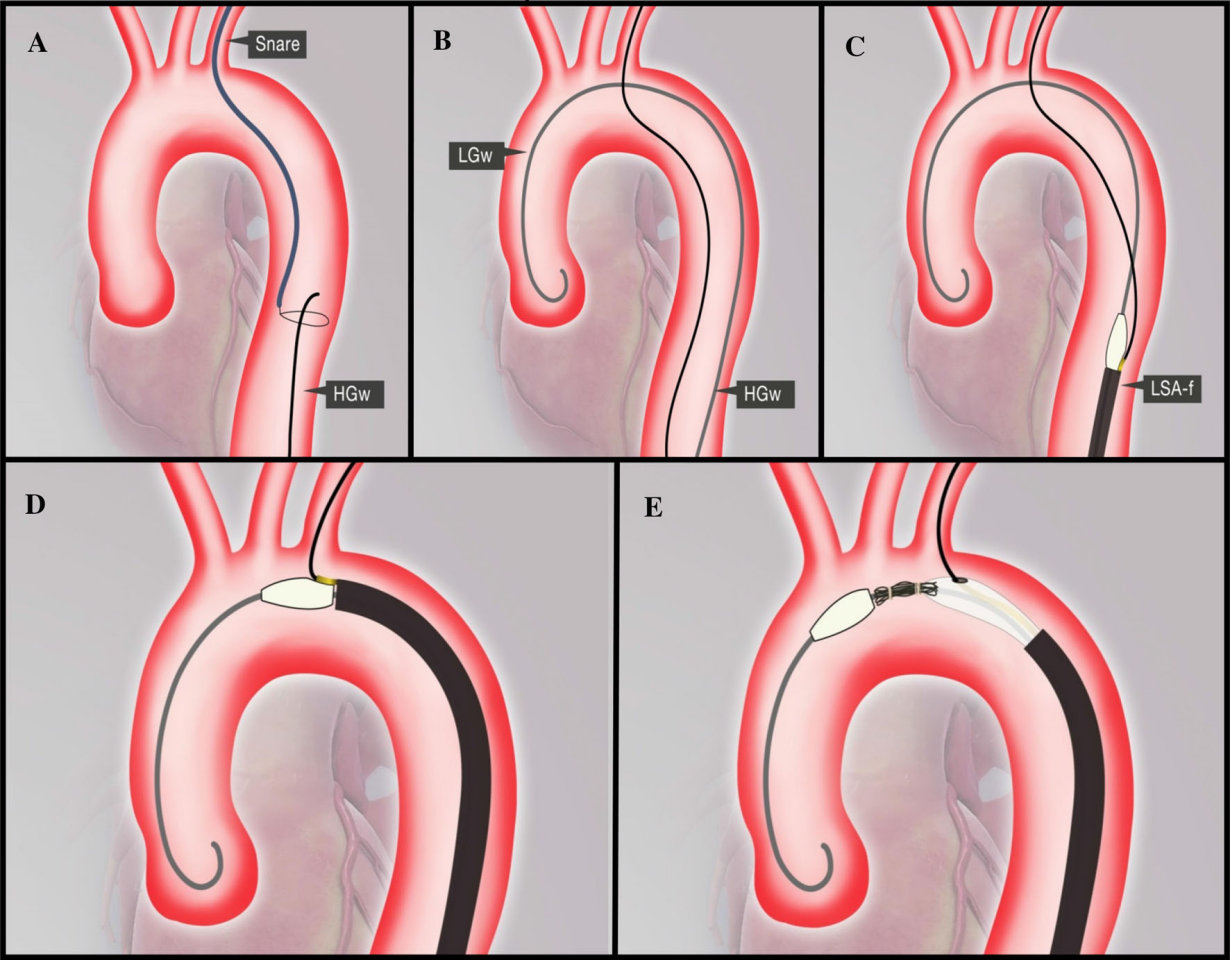
Schematic step by step implantation technique of fenestrated stent graft with two wire system. **A** Hydrophilic guide wire is snared and externalize through left brachial artery. **B** Lunderquist guide wire is placed at the ascending aorta. **C** After loading the Lunderquist guide wire to the central hole and the hydrophilic guidewire to the side hole of the olive tip, the stent graft delivery system is advanced to the descending aorta. **D** Pre-deployment position of the stent graft with the hydrophilic/LSA wire is oriented superiorly. **E** Deployment of the stent graft with the hydrophilic/LSA wire runs from the LSA to the fenestration of the stent graft

The modified stent graft is produced in the factory with standardized design to be off the shelf and will be available in a range of diameters and lengths (Fig. 1A). The first 3 cm is bare metal and the rest is the covered with Dacron. The first radio-opaque marker (Fig. 1A-1) marks the beginning of the covered part of the stentgraft. The second radio-opaque marker (Fig. 1A-2), which is 1 cm distal to the first, marks the site of 10 mm diameter fenestration.

**Fig. 3:**
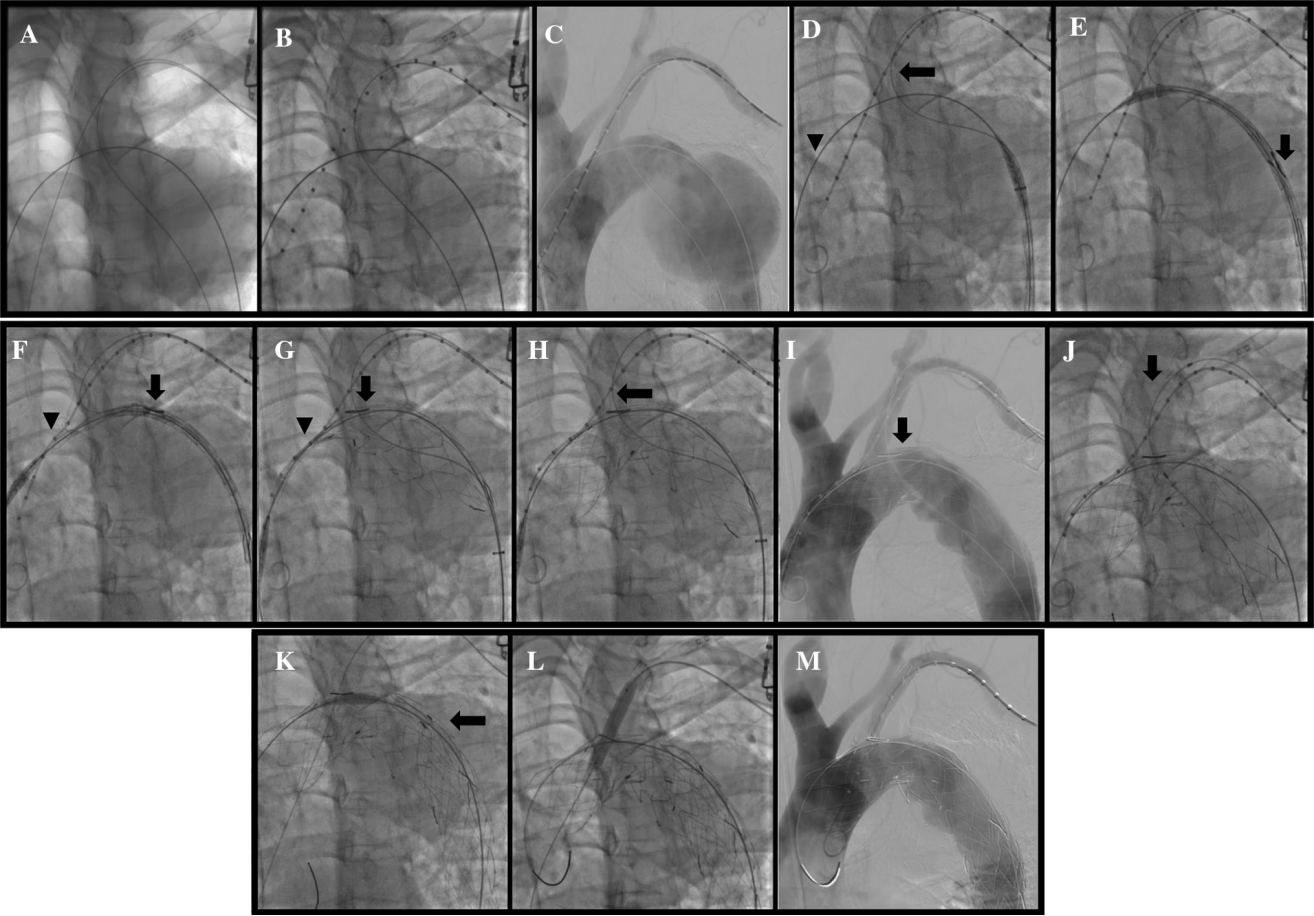
Step by step implantation technique of fenestrated stent graft with two wire system. **A** After surgical arteriotomy of the right femoral artery and percutaneous placement of left brachial sheath (6F Sheath, Terumo, Japan), two guidewires were introduced from the femoral artery: one guide was advanced to ascending aorta (Lunderquist, stiff Guidewire), the second (260 cm, Terumo, Guidewire) was captured and externalized from left brachial artery. The third wire was another Terumo hydrophilic wire placed in asceding aorta from left brachial artery that serves as protective wire and access for pigtail insertion. **B** The measuring pigtail catheter was advanced over the guide wire from left brachial artery. Catheter tip was located at ascending aorta. **C** The aortogram shows large thoracic aortic aneurysm (TAA) in proximal descending aorta, very close to left subclavian artery origin. Anatomic variation in aortic arch: common trunk of right brachiocephalic artery and left carotid artery. The length of zone 2 is about 1 cm. **D** The 34 × 130 mm stent-graft delivery system was advanced over two wires: one is in ascending aorta, another one is in left brachial artery. **E** Fenestration hole marker is clearly seen in the delivery system (arrow). **F** The stent-graft was deployed by pushing the pusher handle while holding firmly the outer sheath until fenestration hole marker was located at left subclavian artery orifice (arrow). Proximal bare stent was captured by delay deploy mechanism (arrowhead). **G** The stent-graft was completely deployed and fenestration hole marker is located exactly at the left subclavian artery orifice (arrow). Proximal bare stent is still captured by delay deploy mechanism (arrowhead). **H** The proximal bare stent was opened by pulling the deployment wire. The side branch wire can be maintained in its position (from femoral artery to left brachial artery through the fenestration with through and through position) during and after stent graft deployment (arrow). **I** Angiography obtained just after stent-graft deployment. Large TAA was completely excluded. Fenestration marker is located slightly distal to the left subclavian orifice (arrow). **J** The subclavian brach stent-graft was advanced from left brachial artery (arrow). **K** An additional stent-graft was placed to make secure distal landing zone overlapping with the first fenestrated stent-graft (arrow). **L** Balloon dilation was performed for the branch stent-graft in the left subclavian artery. **M** The final angiogram showed complete exclusion of TAA and a patent left subclavian artery

The Olive tip has 2 holes: the central hole for the main wire and the side hole for LSA (side branch) wire (Fig. 1C-1, 2 and E-1, 2). The side hole is connected to the fenestration site of the graft by a long, small tube (Fig. 1E-3), which make sure that side branch wire runs into the stent graft via the fenestration and eventually exit the delivery sheath from the side hub at the end of the delivery sheath (Fig. 1F-2). Following femoral access two wires are positioned first in the ascending aorta (Lunderquist guidewire/LGW) and the second into the LSA (hydrophilic guidewire/HGW) by snaring the HGW in the descending aorta and then externalized from the LBA (Fig. 2A, B). The distal ends of the 2 wires are then loaded from the groin into the stent graft through the two holes of the olive tip: the LGW into the central hole (Fig. 1E-1) and the HGW into the side hole (Fig. 1E-2). The 2 wires will then exit the stent graft and delivery sheath trough the main hub (LGW) and side hub (HGW) (Fig. 1D-1, 2 and F-1, 2).

The stent graft is then advanced to the descending aorta over these two wires (Fig. 2C). The separate channel of the LGW and the HGW inside the stent graft and delivery sheath prevents the 2 wires from intertwining, but not anterior/posterior wire trap (described by Joseph G). The later was resolved by retracting the LGW until it is inside the olive tip, rotating the whole system until the wire and the fenestration marker are oriented superiorly and re-advance the LGW to proximal ascending aorta (Fig. 2D).

Partial deployment is performed by pushing the delivery sheath with right hand while the left hand kept the rest of the device stable, releasing the first 7–8 cm of the stent graft to below the fenestration. The markers (Figs. 1E and 3H) can be seen clearly on fluoroscopy and the first marker is positioned just distal to the LCA while the second is positioned at the ostium of the LSA. After checking correct positioning of the stent-graft, the stentgraft is fully unsheathed and released by unscrewing the release mechanism at the hub. Implantation of LSA stent can then be performed through the HGW wire (Fig. 3).

#### Case 1

A 57 years-old male patient had a thoracic aortic aneurysm (TAA) with the maximal diameter of 68 mm and origin of the aneurysm at the lesser curve 8 mm distal to the LSA. The patient was an ex-smoker, suffered from hypertension, type II diabetes and had an ischemic stroke 2 years previously. Two wires were placed; one in ascending aorta (Lunderquist, Cook, MA, USA) and the second into the left brachial artery (Terumo exchange, Terumo, Japan) which was snared from a left brachial puncture (SeQure, Lifetech, Shenzen, China) both from the same right common femoral artery access following a cut down (see Fig. 3 for deployment sequence). The 34 × 130 mm stent-graft delivery system was advanced over the two wires and deployed with the fenestration hole marker located at the origin of the LSA. The LSA diameter measured on CT was 9 mm, so a 10 × 60 mm self expandable expanded polytetrafluoroethylene (e-PTFE) stent-graft (S&G Biotech, Soul, Korea) was advanced and deployed over the wire in the LSA from the femoral access.

**Fig. 4:**
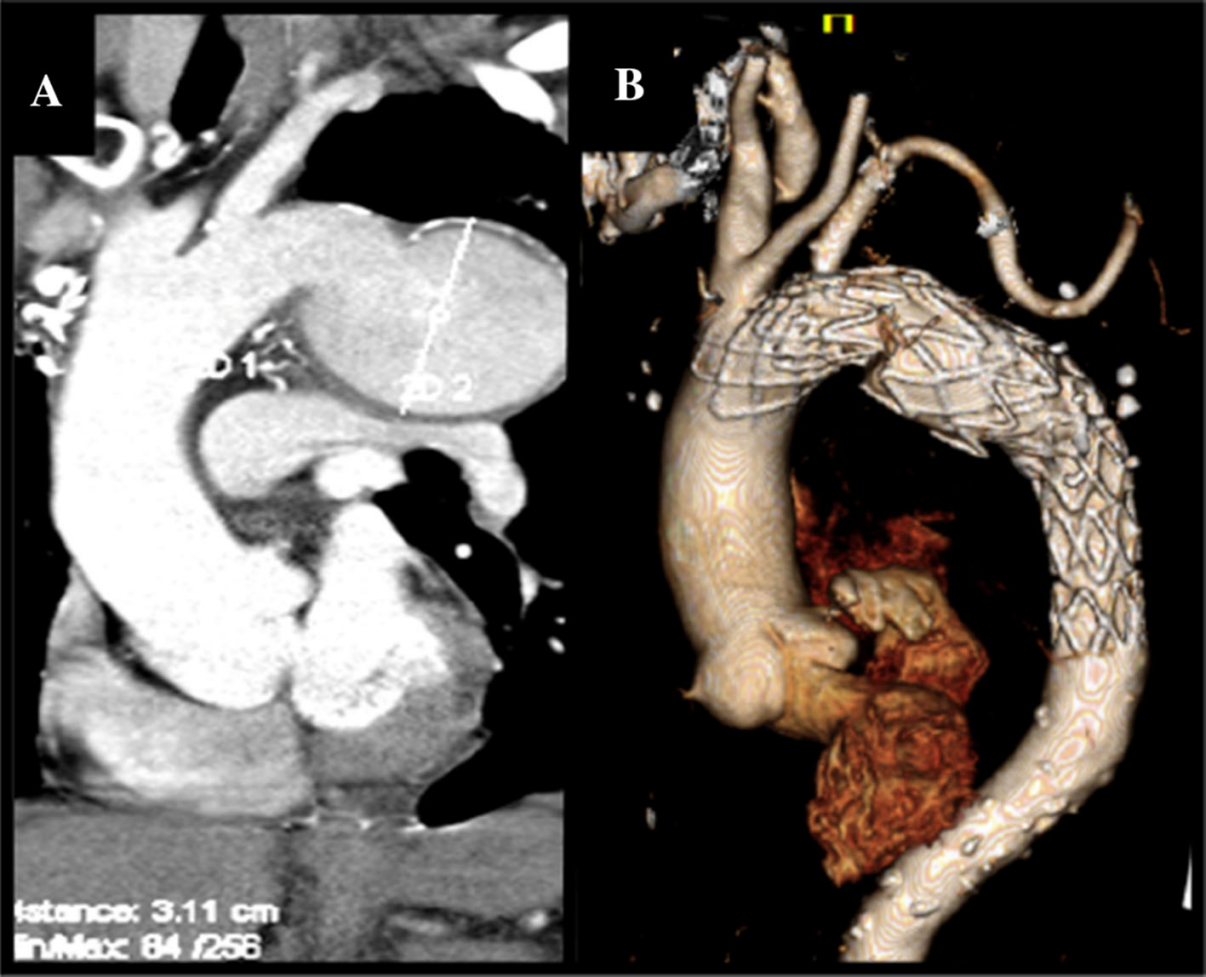
Aortic CT of the first patient before (**A**) and 6 months after the fenestrated TEVAR (**B**). Patency of the LSA stent is maintained after 6 months. No endoleak was observed

The post-implantation aortogram showed complete exclusion of the aneurysm with no endoleak (Fig. 2).There was a moderate stenosis of the LSA stent that was immediately solved by post dilatation of the stent (Mustang 10 × 60 mm, 10 ATM Boston Scientific, USA) (Fig. 3). The total procedure time was 84 min and Fluoroscopic time 32 min. There were no immediate or 30 day complications. Six months follow-up CT showed complete exclusion of the aneurysm, no endoleak, patent LSA stent and slight decrease in maximal diameter of the aneurysm. (65 mm, Fig. 4).

#### Case 2

A 52-year old man presented with an 8 h history acute lower limb ischemia and severe back and abdominal pain to a local hospital. He was a current smoker with uncontrolled hypertension. He was referred to our hospital for urgent revascularization.

On admission, neither femoral artery was palpable, with poor abdominal peristaltic sounds but no peritonitis. Aortic contrast enhanced CT confirmed an aortic dissection with an entry tear 5 mm from the LSA, extending down to the mid abdominal aorta. The false lumen was 24 mm virtually occluding the true lumen at the celiac trunk.

Following informed consent, we performed a fenestrated TEVAR. The stent graft used for this patient was actually prepared for other patient who has an elective schedule, but in view of the greater urgency of this man’s case and similarities in anatomy it was agreed following informed consent to proceed to use the device for this patient instead. After percutaneous puncture of right femoral artery and placement of 7F sheath, we tried crossing the compressed true lumen using an MP-1 (Medtronic, Santa Rosa, CA, USA) angiographic catheter and 260 cm Terumo exchange wire, which failed. Using a percutaneous left brachial artery (LBA) access, a Judkins Right catheter (Medtronic, Santa Rosa, CA, USA) and a 260 cm Terumo exchange wire were advanced to the abdominal aorta. This was then snared (SeQure, Lifetech, Shenzen, China), externalized from the right femoral artery and used to exchange the femoral sheath to an 18F sheath (S&G biotech, Seoul, Korea). Another 260 cm Terumo exchange wire was advanced to the ascending aorta through a MP-1 catheter and exchanged for a super stiff wire (Lunderquist, Cook, MA, USA). Over these two wires, as for case 1, a 32 mm single fenestrated stent-graft was advanced to the distal aortic arch and deployed successfully as previously described (Fig. 3). Subsequently a 10 × 60 mm e-PTFE stent-graft (S&G Biotech, Soul, Korea) was advanced and deployed over the wire in the LSA.

The final aortogram showed complete exclusion of the entry tear, patent LSA, expansion of the true lumen and patent visceral branches. There were no immediate or 30 day complications. Total procedure time was 65 min and fluoroscopic time was 20 min.

**Fig. 5:**
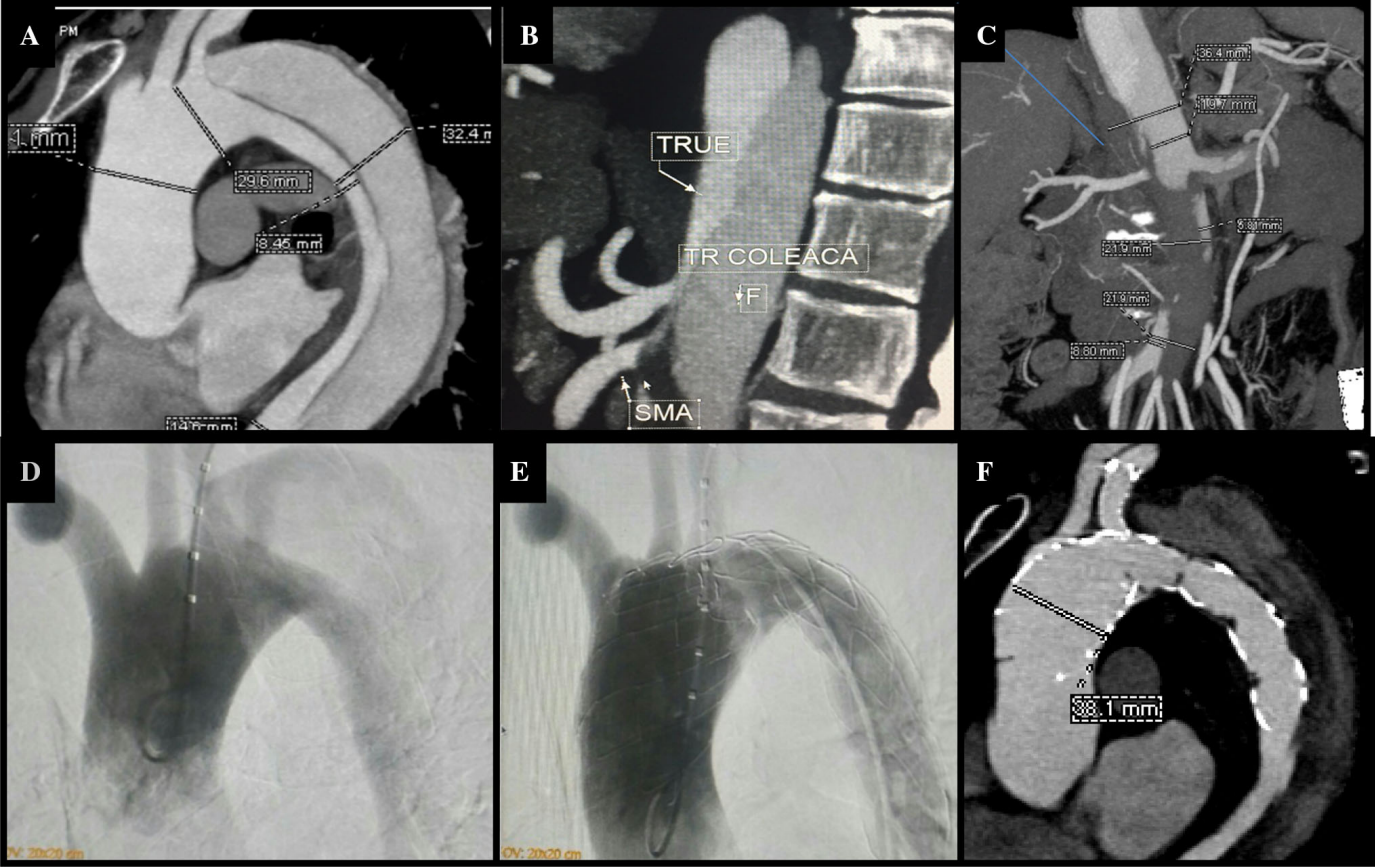
Aortic CT of the second patient showing type B aortic dissection (**A**) with mal-perfusion syndrome due to partial/total occlusion of the celiac trunk, superior mesenteric artery (**B**), renal artery and distal abdominal aorta (**C**). Aortogram before (**D**) and after the fenestrated TEVAR (**E**). The entry tear was located very close to the LSA. After implantation of the fenestrated stent graft, the LSA stent was patent, the entry tear was closed and the false lumen was excluded. 6 months follow up CT showed patent LSA stent and partial thrombosis of the false lumen (**F**)

The patient was discharged at 5 days with resolution of his pain and paralytic ileus as well as improvement of his creatinine levels (2.58–1.4 g/dL). The 6 month CT showed good position of the graft with complete exclusion of the entry tear, no endoleak, partial thrombosis of the false lumen and patent LSA stent (Fig. 5).

## Discussion

There are clearly huge benefits in moving from open to endovascular repair of thoracic aortic disease and increasingly more complex arch and proximal thoracic aortic pathology is being treated in this way.

In this paper we report a new design of a fenestrated stent graft and its first use in man, which we believe will increase the accessibility of fenestrated TEVAR with a less complicated technique. By altering the sequence of fenestration to the side branch, in which wiring of the LSA is performed before rather than after the stent graft is positioned, making deployment simpler and safer. The advancement of the graft on the two wires allows precise positioning to the ostium of the LSA. These guide wires also then allow placement of the branch stent-grafts and balloon dilatations.

During the development of this type of stent graft, Dake and Patel reported on their work on a branched stent graft which has a similar delivery system [13]. The result of the initial clinical trials are encouraging with 100% procedural success and patency of the side branch. However, their device was limited to aneurysms only and not in dissections or emergency cases, as in our second patient (patient 2). Joseph et al. [14] reported their pioneering work on a similar design of a fenestrated stent graft with a preloaded wire that was successfully implanted in two patients with aortic dissections. Similarities include the design of the graft, and the sequence of wiring and deployment. However theirs required an on table fenestration of the stent graft. This is potentially cheaper and upon modification can be perfectly appropriate for patient’s anatomy. However, physician modified stent-grafts lack of quality controls and there is only limited evidence supporting its use. Furthermore, it can take a considerable amount of time for modification of an existing stent-graft before implantation and thus may be an issue in emergency cases.

We believe this new design of stent graft and delivery system offer significant benefits over currently available devices. It has a much simpler deployment because of the newly designed delivery system, which is based on a currently established delivery platform. The separate channel of the LGW and the HGW inside the stent graft and delivery sheath prevents the 2 wires from intertwining that resulted in less step required for implantation. It comes in smaller delivery system (18 F), smaller than the currently available devices (21–24 F OD), which is important especially in an Asian population. The stent graft is produced in the factory with a standardized design and will shortly be available with wide range of diameters and lengths. With this strategy we hope that customization will no longer be needed for single Fenestrated TEVAR and would then possible to be used when the diameter and length is appropriate including in emergency cases. Finally these fenestrated stent-graft are ‘off the shelf’ so they can be used for emergency procedures. They avoid the potential risks of guttering with Chimney stent-grafts and the financial costs of T branch devices, which require much more stock [15].

## Conclusion

In this report, a two wire system fenestrated TEVAR was used in 2 patients with distal aortic arch pathology. It demonstrated a good technical and clinical success using pre-cannulated fenestrations through a newly designed delivery system, which incorporates a modified nose cone olive. We believe this is a major advance over current design in fenestrated and will increase the safety and availability of stent-grafting treatment for aortic arch disease.
